# Astaxanthin activated the SLC7A11/GPX4 pathway to inhibit ferroptosis and enhance autophagy, ameliorating dry eye disease

**DOI:** 10.3389/fphar.2024.1407659

**Published:** 2024-08-19

**Authors:** Chenting Hou, Jie Xiao, Youhai Wang, Xinghui Pan, Kangrui Liu, Kang Lu, Qing Wang

**Affiliations:** ^1^ Department of Ophthalmology, The Affiliated Hospital of Qingdao University, Qingdao, China; ^2^ Eye Hospital of Shandong Province, Jinan, China

**Keywords:** dry eye, ferroptosis, astaxanthin, autophagy, corneal epithelial

## Abstract

Dry eye disease (DED) is a common eye disease in clinical practice. The crucial pathogenesis of DED is that hyperosmolarity activates oxidative stress signaling pathways in corneal epithelial and immune cells and, thus, produces inflammatory molecules. The complex pathological changes in the dry eye still need to be elucidated to facilitate treatment. In this study, we found that astaxanthin (AST) can protect against DED through the SLC7A11/GPX4 pathway. After treatment with AST, the SLC7A11/GPX4 pathway was positively activated in DED both *in vivo* and *in vitro*, accompanied by enhanced autophagy and decreased ferroptosis. In hyperosmolarity-induced DED corneal epithelial cells, AST increased the expression of ferritin to promote iron storage and reduce Fe^2+^ overload. It increased glutathione (GSH) and GPX4, scavenged reactive oxygen species (ROS) and lipid peroxide, and rescued the mitochondrial structure to prevent ferroptosis. Furthermore, inhibition of ferroptosis by ferrostatin-1 (Fer-1), iron chelator deferoxamine mesylate (DFO), or AST could activate healthy autophagic flux. In addition, in a dry eye mouse model, AST upregulated SLC7A11 and GPX4 and inhibited ferroptosis. To summarize, we found that AST can ameliorate DED by reinforcing the SLC7A11/GPX4 pathway, which mainly affects oxidative stress, autophagy, and ferroptosis processes.

## 1 Introduction

Dry eye disease (DED) is a chronic ocular surface disease with high prevalence, affecting the ocular health of 5–50% of the global population (Clayton, 2018). It results in symptoms of discomfort and even ophthalmodynia, caused by tear film instability, ocular surface inflammation and injury, and neuroparesthesia (Luo et al., 2020). The occurrence of dry eye syndrome is related to various factors. Gender differences and hormone regulation play an important role in the ocular surface (Zemanová, 2021). Women are more prone to developing dry eye disease due to cognitive and behavioral differences caused by genetic and environmental factors. People with androgen deficiency and increased prolactin and thyroid-stimulating hormone levels are more susceptible to dry eye disease. Second, looking at the screen for a long time can reduce the blinking time, causing rapid tear evaporation. Deficiencies in vitamins and minerals are also associated with dry eye disease (Zemanová, 2021). The crucial pathogenesis of DED was assumed to be that hyperosmolarity activates oxidative stress signaling pathways in the corneal epithelial cells and produces inflammatory molecules, promoting cell necrosis and apoptosis (Baudouin et al., 2013).

Ferroptosis, regulated by specific cellular mechanisms, is characterized by the accumulation of lipid peroxides in cells catalyzed by excess ferrous irons (Fe^2+^), leading to membrane damage and cell lysis (Dixon et al., 2012; Stockwell et al., 2017). The antioxidative enzymes (such as glutathione peroxidase [GPX4]) can reduce phospholipid hydroperoxide to hydroxyphospholipid, acting as a core inhibitor of reactive oxygen species (ROS) and oxidized lipids (Seibt et al., 2019). GPX4 has been proved to play a crucial role in oxidative homeostasis and cell survival in corneal epithelial cells (Sakai et al., 2016). The continuous expression and function of GPX4 depend on the stable production of intracellular glutathione (GSH) (Ursini and Maiorino, 2020). Cysteine availability is the main limiting factor to synthesize GSH in ferroptosis cells. System xc^−^, consisting of SLC7A11 and SLC3A2, plays a major role in introducing cystine (the oxidized form of cysteine) into cells for subsequent GSH production (Chen X. et al., 2021). Antioxidant inactivation and glutathione depletion lead to cell damage by oxidative stress. Furthermore, the inhibition of ferroptosis by ferrostatin (Fer-1) and deferoxamine mesylate (DFO) is widely found *in vitro* and *in vivo*. Recent studies have shown that Fer-1 activates autophagy and inhibits ferroptosis and, thus, alleviates atherosclerosis (Wu et al., 2021) and liver injury (Totsuka et al., 2019). Fer-1 ameliorates sepsis-induced cardiac dysfunction and alleviates cardiac ferroptosis and inflammation, possibly by inhibiting the TLR4/NF-κB signaling pathway (Coursey et al., 2016).

Ferroptosis has been clarified in various pathological and physiological conditions, including cancer therapy (Liang et al., 2019), neurodegenerative diseases (Do Van et al., 2016), myocardial ischemia–reperfusion injury (Wu et al., 2021), and age-related macular degeneration (Totsuka et al., 2019). The majority of previous studies have investigated cell necrosis, apoptosis (Coursey et al., 2016), and pyroptosis (Chen et al., 2020) as the partial mechanism of DED in response to pro-oxidants. The latest literature shows that ferroptosis induced by oxidative stress is involved in DED (Zuo et al., 2022).

Astaxanthin (AST), which is known as “super vitamin E,″ is a potent ketocarotenoid antioxidant that exhibits strong anti-inflammatory properties, attracting considerable interest due to its various biological effects, including antioxidation, neuroprotection, anti-hyperplasia, anti-inflammation, anti-apoptosis (Yuan et al., 2011; Galasso et al., 2018), and even anti-ferroptosis properties in several diseases, which are different to those of vitamin E (Kong et al., 2023; Ren et al., 2023). Astaxanthin can activate GPX4 to inhibit ferroptosis and reduce metal-induced biotoxicity (Li et al., 2024). Oral products containing astaxanthin can improve tear film stability and relieve dry eye symptoms (Huang et al., 2016). The anti-inflammatory system–PI3K/Akt signaling pathway has been suggested as the partial potential mechanism of AST on DED via downregulating the expression of HMGB1 (Li et al., 2020). However, the protective effects of AST against ferroptosis in DED remain unclear. In the present study, we established a dry eye model *in vitro* and *in vivo* to elucidate whether AST inhibits ferroptosis via regulating SLC7A11/GPX4 and activates autophagy to protect against DED. Combined with the previous results, our findings will broaden the understanding of DED and the relationship between autophagy and ferroptosis, thus providing a new therapeutic target for the treatment of DED.

## 2 Methods

### 2.1 Cell culture and treatment

Human corneal epithelial cells (HCECs), a human SV40 immortalized corneal epithelial cell line (CRL-11135, HCE-2; ATCC, Manassas, VA, United States), between passages 15 and 20, were cultured as previously described. The immortalized HCECs were then treated with different osmolarities, ranging from 312 to 550 mOsM, which was achieved by adding 0, 70, 90, or 120 mM sodium chloride (NaCl) (Sigma-Aldrich, St. Louis, MO, United States). Lipid ROS scavenger Fer-1 (MCE, Shanghai) and iron chelator DFO (MCE, Shanghai) were used as cell death inhibitors 4 h prior to NaCl exposure. Dimethyl sulfoxide (DMSO; Wako) was used as the vehicle and the control for AST and Fer-1 treatments. DFO and Fer-1, regarded as known ferroptosis inhibitors, were used to further confirm the presence of ferroptosis and as a reference to further illustrate the anti-ferroptosis effect of astaxanthin.

### 2.2 Animal model and treatment

In this study, 6–8-week-old female BALB/c mice (Pengyue Laboratory, Jinan, China) were used for this study. All animals were maintained according to the Association for Research in Vision and Ophthalmology (ARVO) statement for the Use of Animals in Ophthalmic and Vision Research. The mice were randomly divided into four groups as follows (*n* = 5): 1) control, 2) DE, 3) DMSO + DE, and 4) AST + DE. DE indicates the dry eye group. Astaxanthin was dissolved in DMSO to obtain a mixture of 10 μM. The mice in groups 2, 3, and 4 were subcutaneously injected with 0.5 mL of scopolamine hydrobromide (2.5 mg/mL; MedChemExpress) three times a day for 14 days to induce ocular surface injury. Among them, groups 3 and 4 received 1 μL AST and DMSO, respectively, four times a day in both eyes. The eyes were immediately removed from each animal, some of which were processed with paraformaldehyde and stored at 4°C for tissue embedding and sectioning. Cornea tissues were stored at −80°C, and homogenates were processed with formaldehyde and glutaraldehyde for protein and histological analysis. All mice were euthanized through quick cervical dislocation. The experiments were performed at least three times to verify the reproducibility of the result.

### 2.3 Cell survival activity assay

Cells were seeded and grown in 96-well plates for various treatments. Cell viability was assessed using the Cell Counting Kit-8 (CCK-8; DOJINDO, Kumamoto, Japan) assay. Then, 10 μL of the CCK-8 solution was added to the 100-μL fresh medium. Absorbance was measured at 450 nm after 2 h of incubation in the cell incubator.

### 2.4 Western blot analysis

Corneas and cells were lysed with RIPA lysis buffer (Solarbio, Beijing, China). Protein concentrations were measured by BCA assay and mixed with SDS sample buffer (1:1) and boiled for 10 min at 95°C. The protein samples were separated by 10%–12% sodium dodecyl sulfate–polyacrylamide gel electrophoresis (SDS-PAGE) and transferred onto a polyvinylidene difluoride (PVDF) membrane. The membranes were blocked with 5% skimmed milk at room temperature for 2 h and then incubated with a primary antibody diluted by antibody dilution buffer (Beyotime, Shanghai, China) at 4°C overnight. Primary antibodies included the antibody to GPX4 (Abmart, Shanghai, China), ferritin (Abmart, Shanghai, China), SLC7A11 (Abmart, Shanghai, China), LC3 (CST, United States), P62 (CST, United States), and GAPDH (CST, United States). The following day, the membranes were washed with 1 × Tris-buffered saline and Tween 20 (TBST) three times and incubated with secondary antibodies at room temperature for 1 h. Protein expression levels were tested with chemiluminescence assay (Thermo Fisher Scientific, Waltham, MA, United States). The ratio of the gray value of the target band to GAPDH was representative of the relative protein expression.

### 2.5 RNA isolation and quantitative real-time PCR

Total RNA was extracted from HCECs using the TRIzol reagent (Vazyme Biotech Co., Ltd., Nanjing, China), according to the manufacturer’s instructions. The RNA was reverse-transcribed to cDNA using a HiScript^®^ III Reverse Transcription SuperMix for qPCR (Vazyme, Nanjing, China), and then, cDNA was used for PCR using the ChamQ Universal SYBR qPCR Master Mix (Vazyme, Nanjing, China). The relative expression of RNA was normalized to the endogenous control GAPDH using the 2^−ΔΔCT^ method (Pan et al., 2023). The primers used in this study are as follows:

**Table:** 

	Forward	Reverse
TFRC	5′-TTT​CCA​CCA​TCT​CGG​TCA​TC-3′	5′-GCT​TCA​CAT​TCT​TGC​TTT​CTG​AG-3ʹ
FPN	5′-GCA​GGA​GAA​GAC​AGA​AGC​AAA​C-3′	5′-AAA​TAA​AGC​CAC​AGC​CGA​TGA​C-3′
HEPH	5′-CAG​TTA​TGG​TTA​CAT​TTT​CCT​GAG​C-3′	5′-GGA​CCC​AAG​ATT​CCC​AAG​TG-3′
FTH1	5′-TCC​TAC​GTT​TAC​CTG​TCC​ATG​T-3′	5′-GTT​TGT​GCA​GTT​CCA​GTA​GTG​A-3′
FTL1	5′-TAC​GAG​CGT​CTC​CTG​AAG​ATG​C-3ʹ	5′-GGT​TCA​GCT​TTT​TCT​CCA​GGG​C-3′
DMT1	5′-GGC​TTT​CTT​ATG​AGC​ATT​GCC​TA- 3′	5′-GGA​GCA​CCC​AGA​GCA​GCT​TA-3′
CP	5′-TTT​CCT​GCT​ACC​CTG​TTT​GAT​G-3′	5′-CGG​CTT​TCA​GAT​GGT​TTA​GAT​TC-3′
NCOA4	5′-ACA​GTT​GCA​TAA​GCC​GTC​ACC-3′	5′-TGA​GCC​TGC​TGT​TGA​AGT​GTC-3′
STEAP3	5′-CAG​CCC​TAT​GTG​CAG​GAA​AG-3′	5′-GCA​AGT​ACA​CGA​GTG​ACA​GCA-3′
GAPDH	5′-CAA​CGT​GTC​AGT​GGT​GGA​CCT​G-3′	5′-GTG​TCG​CTG​TTG​AAG​TCA​GAG​GAG-3′

### 2.6 Tissue immunofluorescence

Mouse eye sections were fixed with 4% paraformaldehyde in PBS for 15 min, then permeabilized with 0.5% Triton X-100 for 15 min, and blocked with 10% goat serum for 1 h. The samples were stained with a rabbit monoclonal anti-GPX4 antibody (1:250; Abmart) in antibody dilution buffer (Beyotime, Shanghai, China) at 4°C overnight. Secondary staining was performed with Alexa Fluor^TM^ 488 goat anti-rabbit IgG (1:200; Invitrogen, United States) and Alexa Fluor^TM^ 555 goat anti-rabbit IgG (1:200; Invitrogen, United States) for 1 h at room temperature, and the nuclei were counterstained with DAPI for 7 min.

### 2.7 Fe^2+^ detection

Intracellular Fe^2+^ was assessed by treatment with 5 μM FeRhoNox-1 (Goryo Chemical, Inc., Sapporo, Japan) in Hank’s balanced salt solution (HBSS; Thermo Fisher Scientific). After washing, the cells were observed under a fluorescence microscope (BZ-9000; Keyence). At least four fields per chamber were imaged, and the fluorescence intensities were analyzed using ImageJ (https://imagej.nih.gov/ij/; provided in the public domain by the National Institutes of Health, Bethesda, MD, United States).

### 2.8 Intracellular glutathione

HCECs seeded into six-well plates were used. Cells digested by trypsin were collected and washed twice with PBS, and a reagent was added (GSSG/GSH quantification kit). First, the cells were resuspended and then lysed by repeated freezing and thawing 2–3 times. Then, the cells were centrifuged, and the supernatant was collected at 4°C for measurement. The total protein concentration was detected using the Pierce bicinchoninic acid (BCA) Protein Assay Kit (Thermo Fisher Scientific, Shanghai, China), and the GSH concentration was analyzed using the GSSG/GSH quantification kit (GSH assay; Solarbio). The corrected GSH content of the samples is the ratio of the supernatant concentration to protein concentration obtained using a 96-well plate according to the manufacturer’s protocol, and a microplate reader was used to measure absorbance at 412 nm.

### 2.9 Reactive oxygen species detection

HCECs were seeded into 96-well plates and pre-treated with 10 μM Fer-1, 100 μM DFO, or 10 μM AST for 4 h and then with 120 mM NaCl for 24 h. Intracellular ROS was determined using a Reactive Oxygen Species Assay Kit (Solarbio, CA1410) through the DCFH-DA ROS probe, according to the manufacturer’s instructions. After washing, adherent cells were observed under a fluorescence microscope. The collected suspended cells were detected using the FL1 channel of flow cytometry and then analyzed using FlowJo v10.6.2.

### 2.10 MDA kit

The MDA amount often reflects the degree of lipid peroxidation in the body, which indirectly indicates the degree of cell damage. The brain tissues or cells were mixed using normal saline in a ratio of 1:9 and placed on ice. After homogenization, the brain tissues were centrifuged at 12,000 rpm at 4°C for a quarter. The MDA content was detected following the manufacturer’s protocol (Nanjing Jiancheng Bioengineering Institute, Nanjing, China) (Du et al., 2022; Yu et al., 2022). The absorbance at 532 nm was assessed using a microplate reader (Thermo Fisher Scientific, United States).

### 2.11 Transmission electron microscopy

HCECs were cultured and stimulated according to experimental needs. At a confluent density of 90%, HCECs were harvested and fixed with 2.5% glutaraldehyde (Servicebio, China) for 30 min at room temperature and stored at 4°C. The cell mass after centrifugation was then rinsed three times with 0. 1M PB (pH 7.4), and the fixed HCECs were post-fixed using 2% osmium tetroxide for 2 h at room temperature. After washing with PB three times, the cells were extracted and suspended in 1% agarose solution to be solidified for pre-embedding. After washing another time, the cells were dehydrated in a graded series of alcohol (30%, 50%, 70%, 80%, 90%, 95%, and 100%) before being embedded in EMBed 812 (SPI, United States), and ultrathin sections (60–80 nm) were prepared using a Leica ultramicrotome (Leica Microsystems, United States) and fished out onto the 150-mesh cuprum grids with a formvar film. The prepared sections were then subjected to double staining with uranyl acetate and lead citrate. The cuprum grids were observed under a transmission electron microscope (TEM; HT7800, Hitachi, Japan), and the images were obtained.

### 2.12 Statistical analyses

Data are presented as the mean ± standard error of the mean (SEM). All statistical analyses were performed using the GraphPad Prism 7.0 program (GraphPad Software Inc., San Diego, CA). Values with *p* < 0.05 were considered statistically significant. The quantitative experiments were all repeated three times.

## 3 Results

### 3.1 Hyperosmolarity causes abnormal iron homeostasis in HCECs

**FIGURE 1:**
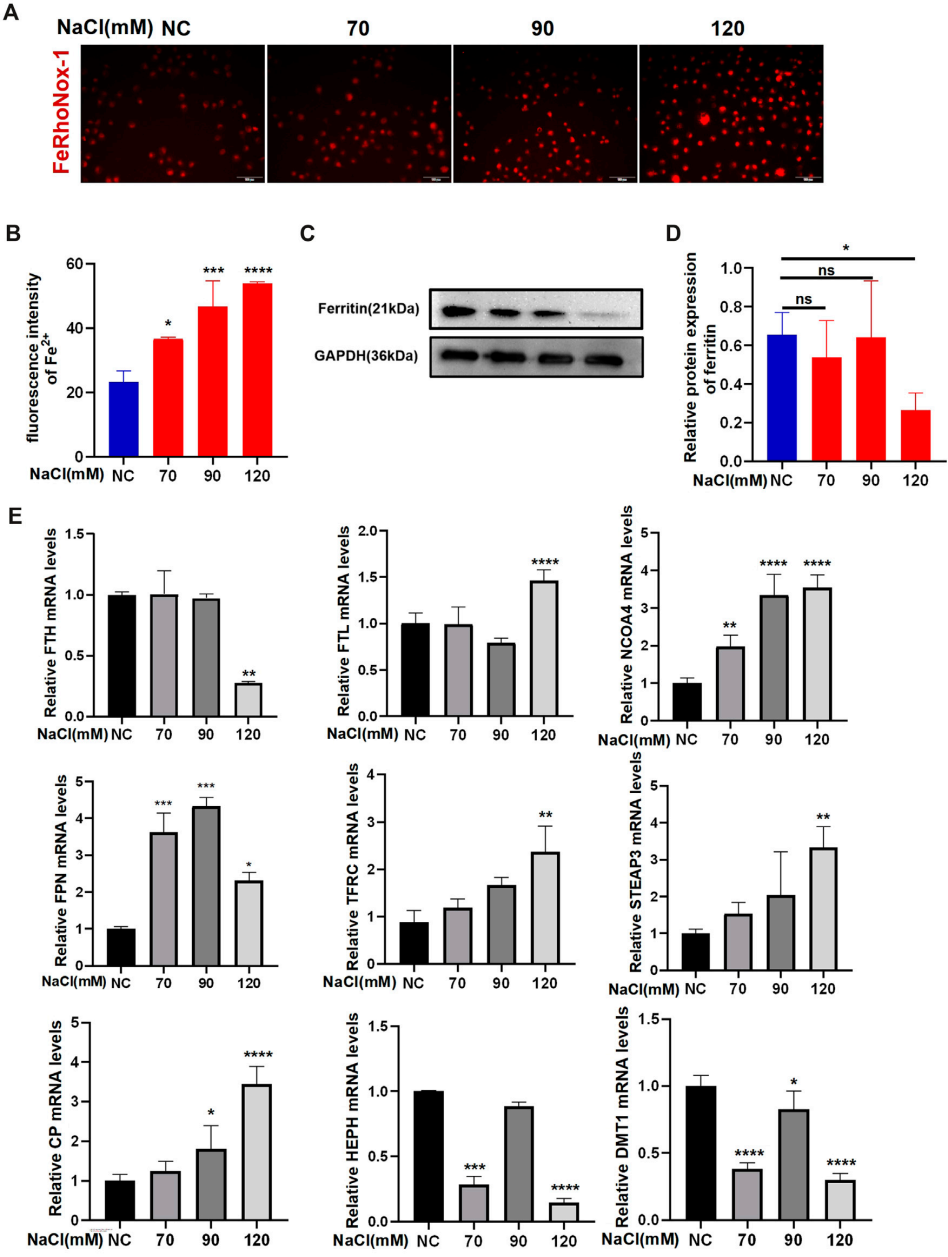
Hyperosmolarity disrupts iron homeostasis in HCECs. **(A,B)** Western blot analysis of ferritin in HCECs after various concentrations of NaCl for 24 h; protein levels of ferritin were normalized to those of GAPDH. **(C)** Quantification of fluorescence intensity of Fe^2+^ specifically detected by FeRhoNox-1 in HCECs. **(D)** Intracellular Fe^2+^, 24 h after incubating cells with serial concentrations of NaCl (0, 70, 90, and 120 mM), was visualized by 5 μM FeRhoNox-1 staining coupled with confocal microscopy. Scale bar: 100 μm. **(E)** qRT-PCR analysis of iron homeostasis-related genes in HCECs exposed to various concentrations of NaCl for 24 h. ns, not significant. *p < 0.05, **p < 0.01, ***p < 0.001, and ****p < 0.0001.

Imaging of Fe^2+^ using FeRhoNox-1 showed that hyperosmolarity significantly enhanced intracellular Fe^2+^ levels in HCECs at 24-h exposure. The increase in Fe^2+^ levels in HCECs caused by hyperosmolarity is concentration-dependent (Figures 1A, B). qRT-PCR and Western blotting were used to analyze the expression of ferroptosis-related genes and proteins, respectively. Immunoblotting analysis indicated that 120 mM NaCl elicited a significant decrease in protein levels of ferritin (Figures 1C, D). Transferrin (TF) traps ferric iron (Fe^3+^) and binds to protein TF receptor 1 (TFR1) on the cell membrane to endocytose Fe^3+^. Fe^3+^ is then reduced to Fe^2+^ by a six-transmembrane epithelial antigen of the prostate 3 (STEAP3), and divalent metal transporter 1 (DMT1) delivers Fe^2+^ from endosomes to the cytoplasm. Partial ferric iron is stored in the ferritin, which consists of ferritin light chain (FTL) and ferritin heavy chain 1 (FTH1). Ferritinophagy modulated by a cargo protein called nuclear receptor coactivator 4 (NOCA4) causes ferritin degradation and thereby releases Fe^2+^. Ferroportin (FPN) acts synergistically with ferroxidase ceruloplasmin (CP) or hephaestin (HEPH) to export excessive Fe^2+^ out of the cells. The mRNA levels of FPN, TFRC, FTL, STEAP3, and CP were significantly upregulated by 120 mM NaCl for 24 h. NCOA4 mRNA levels were upregulated by 90 or 120 mM NaCl. Hyperosmolarity downregulated the mRNA levels of FTH, HEPH, and DMT1 (Figure 1E).

### 3.2 Hyperosmolarity stimulates ferroptosis in HCECs

**FIGURE 2:**
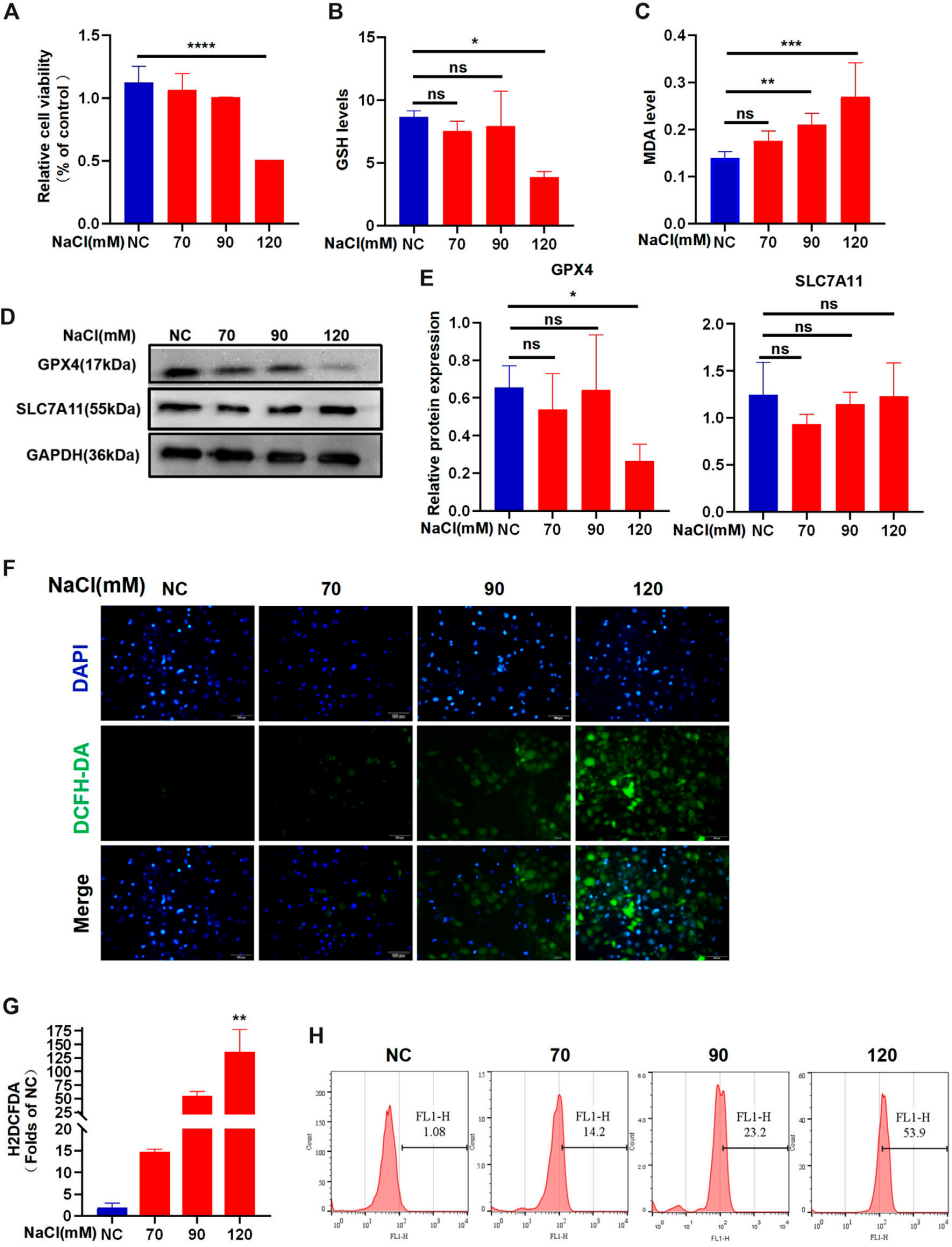
Hyperosmolarity induces ferroptosis in HCECs. **(A)** Cell viability 24 h after incubating HCECs with serial concentrations of NaCl (0, 70, 90, and 120 mM), was probed by CCK-8 assay. **(B)** GSH levels in HCECs after various concentrations of NaCl for 24 h were determined using a GSH assay kit. **(C)** MDA levels after various concentrations of NaCl for 24 h were detected using an MDA assay kit. **(D,E)** Western blot analysis of SLC7A11 and GPX4 in HCECs after various concentrations of NaCl for 24 h; protein levels of SLC7A11 and GPX4 were normalized to those of GAPDH. **(F)** Intracellular ROS generation was visualized using the fluorescent probe DCFH-DA by fluorescence microscopy. Nuclei were stained with DAPI (blue). **(G)** Levels of ROS in HCECs treated with various concentrations of NaCl for 24 h were assessed by DCFH-DA staining coupled with flow cytometry. **(H)** Quantification of fluorescence intensity acquired by flow cytometry. Values are shown as the mean ± SD. ns, not significant; *p < 0.05, **p < 0.01, and ***p < 0.001.

The data from the CCK-8 assay showed that 120 mM NaCl for 24 h caused lower cell viability (Figure 2A). Intracellular GSH levels showed a decreasing trend when exposed to NaCl at a concentration of 70–120 mM and indicated statistical significance at 120 mM (Figure 2B). Hyperosmolarity significantly increased lipid peroxide MDA levels in HCECs in a concentration-dependent manner (Figure 2C). Immunoblot analysis showed that the relative protein level of SLC7A11 was not changed. In contrast, the protein expression of GPX4 in HCECs was significantly downregulated by treating with 120 mM NaCl for 24 h (Figures 2D, E). Intracellular ROS generation visualized using the fluorescent probe H2DCFDA by fluorescence microscopy showed that total ROS levels were increased at 90 mM NaCl and substantially elevated at 120 mM NaCl (Figure 2F). Fluorescence quantization and flow cytometry yielded the same results (Figures 2G, H).

### 3.3 Suppression of ferroptosis by Fer-1 protects HCECs against hyperosmolarity exposure

**FIGURE 3:**
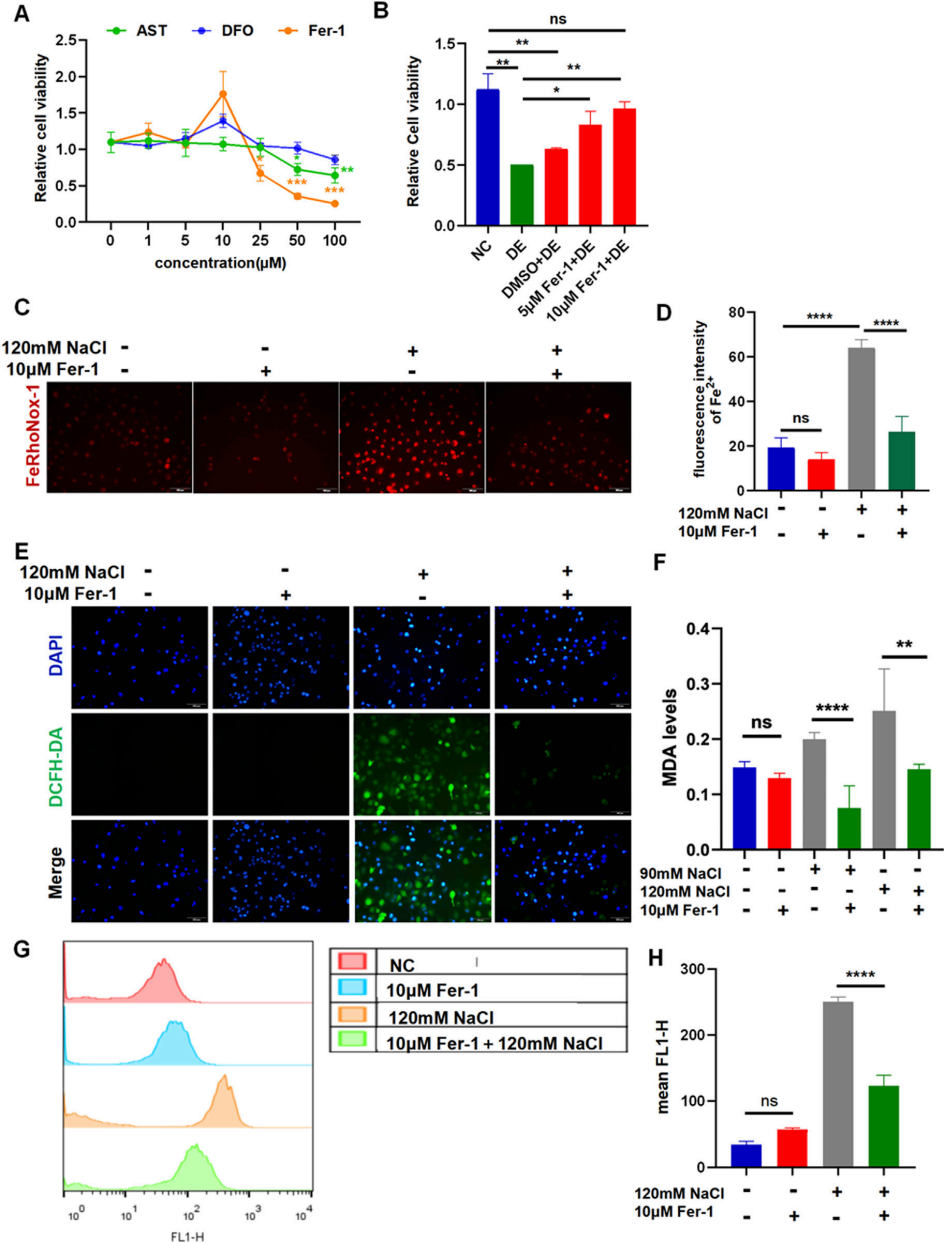
Ferroptosis inhibitor Fer-1 relieves hyperosmolarity-induced ferroptosis in HCECs. **(A)** Cell viability was determined by CCK-8 assay after pretreating with serial concentrations of Fer-1, DFO, or AST for 24 h. **(B)** HCEC viability was assessed after pretreating with 5 μM or 10 μM Fer-1 for 6 h and then incubated for 24 h with 120 mM NaCl (DE indicates the dry eye group, treated with 120 mm NaCl). **(C)** Quantification of fluorescence intensity of Fe^2+^ detected by FeRhoNox-1. **(D)** Cells were pretreated with 10 μM Fer-1 for 6 h and incubated with 120 mM NaCl for 24 h. Intracellular Fe^2+^ was stained with 5 μM FeRhoNox-1 and observed by microscopy. Scale bar: 100 μm. **(E)** Intracellular ROS was visualized using a fluorescent probe DCFH-DA and fluorescence microscopy. Nuclei were stained blue with DAPI. Scale bar: 100 μm. **(F)** MDA levels were detected in HCECs, which were pretreated with 10 μM Fer-1 for 6 h and then incubated with 90 or 120 mM NaCl for 24 h. **(G)** Levels of ROS were assessed by DCFH-DA staining coupled with flow cytometry. **(H)** Quantification of fluorescence intensity acquired by flow cytometry. ns, not significant; *p < 0.05, **p < 0.01, ***p < 0.001, and ****p < 0.0001.

The results of the CCK-8 assay revealed that at a concentration of 1 μM–100 μM, the three chemicals begin to cause a decrease in cell viability to preliminarily determine the intervention concentration (Figure 3A). The treatment with AST at concentrations of 50 μM and 100 μM significantly decreased the cell viability in a concentration-dependent manner. The corneal epithelial cell viability remarkably reduced by Fer-1 at concentrations of 25 μM, 50 μM, and 100 μM. The intervention with DFO at 100 μM maintains constant cell viability (Figure 3A). The cells were pretreated with the three reagents for 6 h before incubation with NaCl. The viability of HCECs, following 24-h exposure to 120 mM NaCl, was significantly increased from 50.51% to 83.26% by 5 μM and from 50.51% to 96.73% by 10 μM Fer-1 (Figure 3B). So, the intervention concentration of Fer-1 to inhibit ferroptosis was 10 μM. Fe^2+^ production caused by 120 mM NaCl was significantly attenuated by 10 μM Fer-1 in HCECs (Figures 3C, D). Moreover, it also inhibited intracellular ROS fluorescence intensity detected by a microscope and flow cytometry (Figures 3E–G). Similarly, the MDA levels indicated that the accumulation of lipid peroxidation in HCECs caused by treatment with either 90 mM or 120 mM NaCl for 24 h was significantly cleared by 10 μM Fer-1 (Figure 3F). These findings imply that Fer-1 attenuates ferroptosis induced by hyperosmolarity in HCECs.

### 3.4 Inhibiting ferroptosis by DFO in HCECs

**FIGURE 4:**
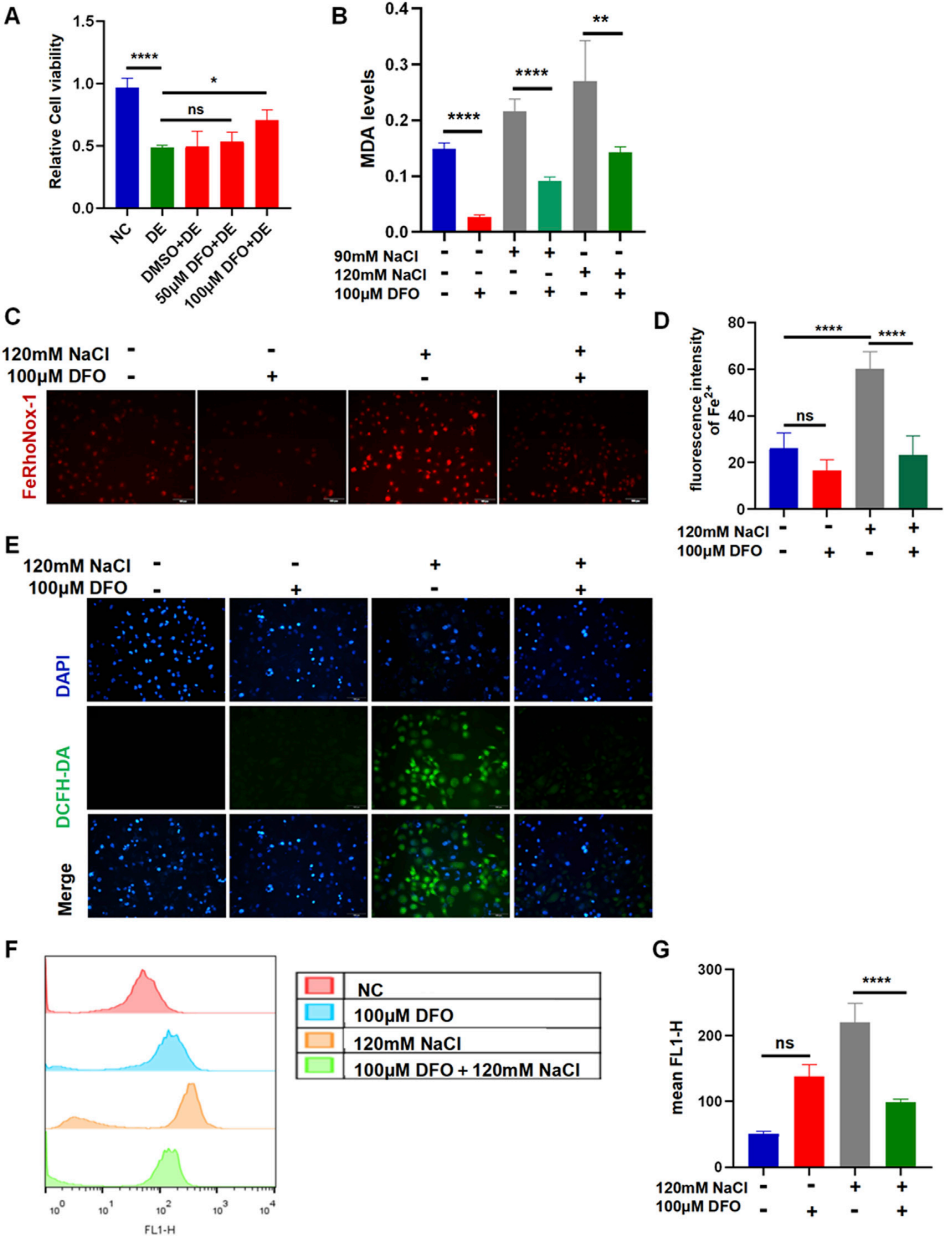
Iron-chelating agent DFO relieves hyperosmolarity-induced ferroptosis in HCECs. **(A)** HCEC viability was assessed after pretreating with 50 μM or 100 μM DFO for 6 h and then incubated for 24 h with 120 mM NaCl (DE indicates the dry eye group, treated with 120 mm NaCl). **(B)** MDA levels were detected using an MDA assay kit. HCECs were pretreated with 100 μM DFO for 6 h and then incubated with 90 mM or 120 mM NaCl for 24 h. **(C)** Intracellular Fe^2+^ was observed by fluorescence microscopy with 5 μM FeRhoNox-1. Scale bar: 100 μm. **(D)** Quantification of fluorescence intensity of Fe^2+^ detected by FeRhoNox-1. **(E)** Intracellular ROS was visualized using the probe DCFH-DA. HCECs were pretreated with 100 μM DFO for 6 h and then incubated with 120 mM NaCl for 24 h. Nuclei were stained blue with DAPI. Scale bar: 100 μm. **(F)** Levels of ROS were stained by DCFH-DA with flow cytometry. **(G)** Quantification of fluorescence intensity acquired by flow cytometry. ns, not significant; *p < 0.05, **p < 0.01, ***p < 0.001, and ****p < 0.0001.

The viability of HCECs after 24 h of incubation with 120 mM NaCl was still increased from 48.93% to 70.84% by 100 μM DFO (Figure 4A). The MDA levels indicated that the accumulation of lipid peroxidation in HCECs caused by treatment with either 90 mM or 120 mM NaCl for 24 h was significantly reduced by 100 μM DFO (Figure 4B). Fluorescence microscopy with FeRhoNox-1 staining revealed that 100 μM DFO significantly decreased the levels of Fe^2+^ induced by 120 mM NaCl (Figures 4C, D). As expected, 100 μM DFO significantly inhibited the production of ROS in HCECs after 24 h of exposure to 120 mM NaCl (Figure 4E). Moreover, we also used flow cytometry to quantify ROS in cells exposed to 120 mM NaCl and found that it was significantly reduced by 100 μM DFO (Figures 4F, G).

### 3.5 AST protects HCECs from ferroptosis caused by hyperosmolarity via the SLC7A11/GPX4 signaling pathway

**FIGURE 5:**
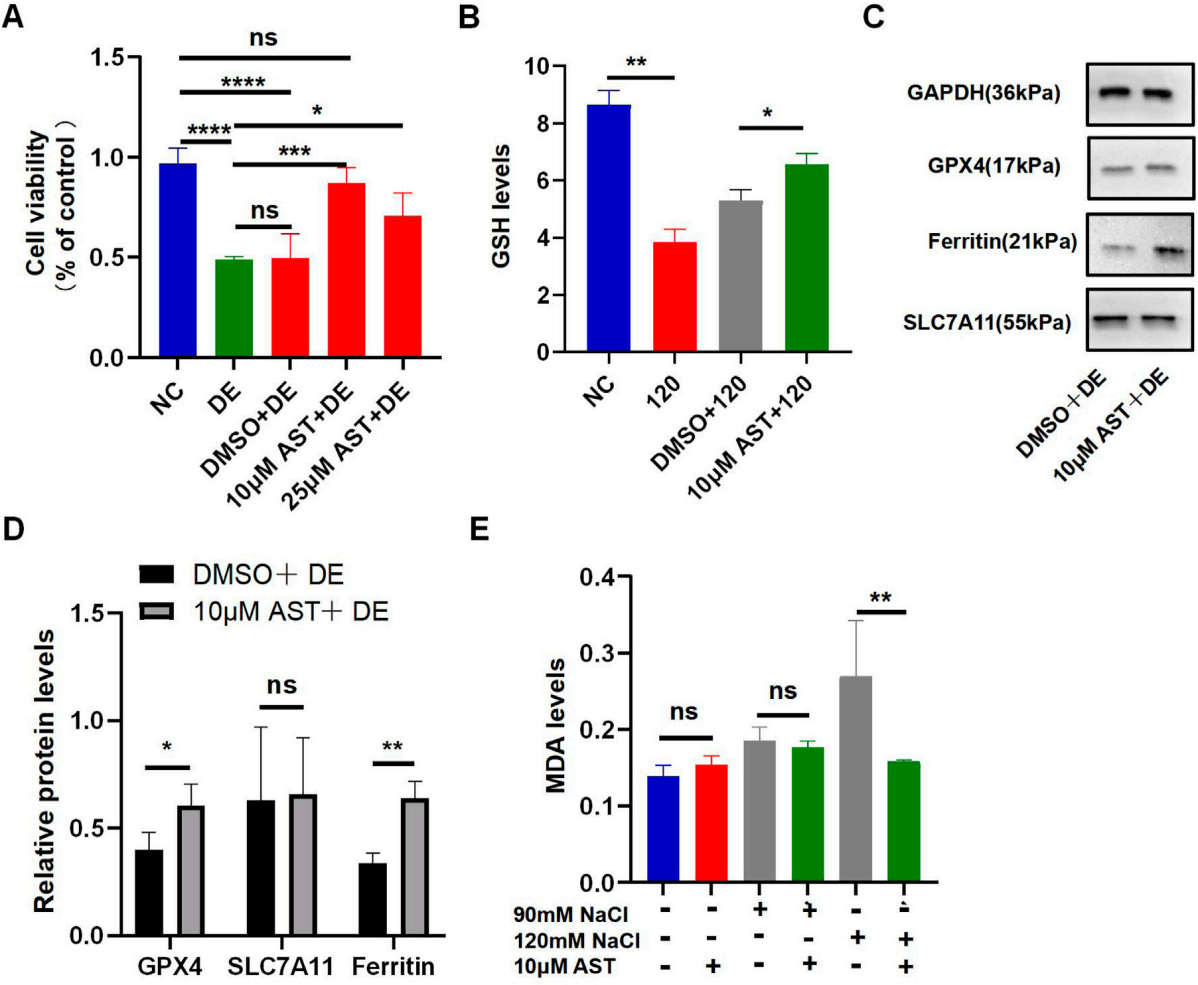
AST relieved hyperosmolarity-induced ferroptosis in HCECs. **(A)** HCEC viability was assessed after pretreating with 10 μM or 25 μM AST for 6 h and then incubated for 24 h with 120 mM NaCl. (DE indicates the dry eye group, treated with 120 mm NaCl). **(B)** GSH levels were detected using a GSH assay kit. **(C,D)** Western blot analysis of SLC7A11, GPX4, and ferritin in HCECs after pretreating with DMSO or 10 μM AST for 6 h and then incubated for 24 h with 120 mM NaCl; protein levels were normalized to those of GAPDH. **(E)** MDA levels were detected using an MDA assay kit. ns, not significant; *p < 0.05, **p < 0.01, ***p < 0.001, and ****p < 0.0001.

**FIGURE 6:**
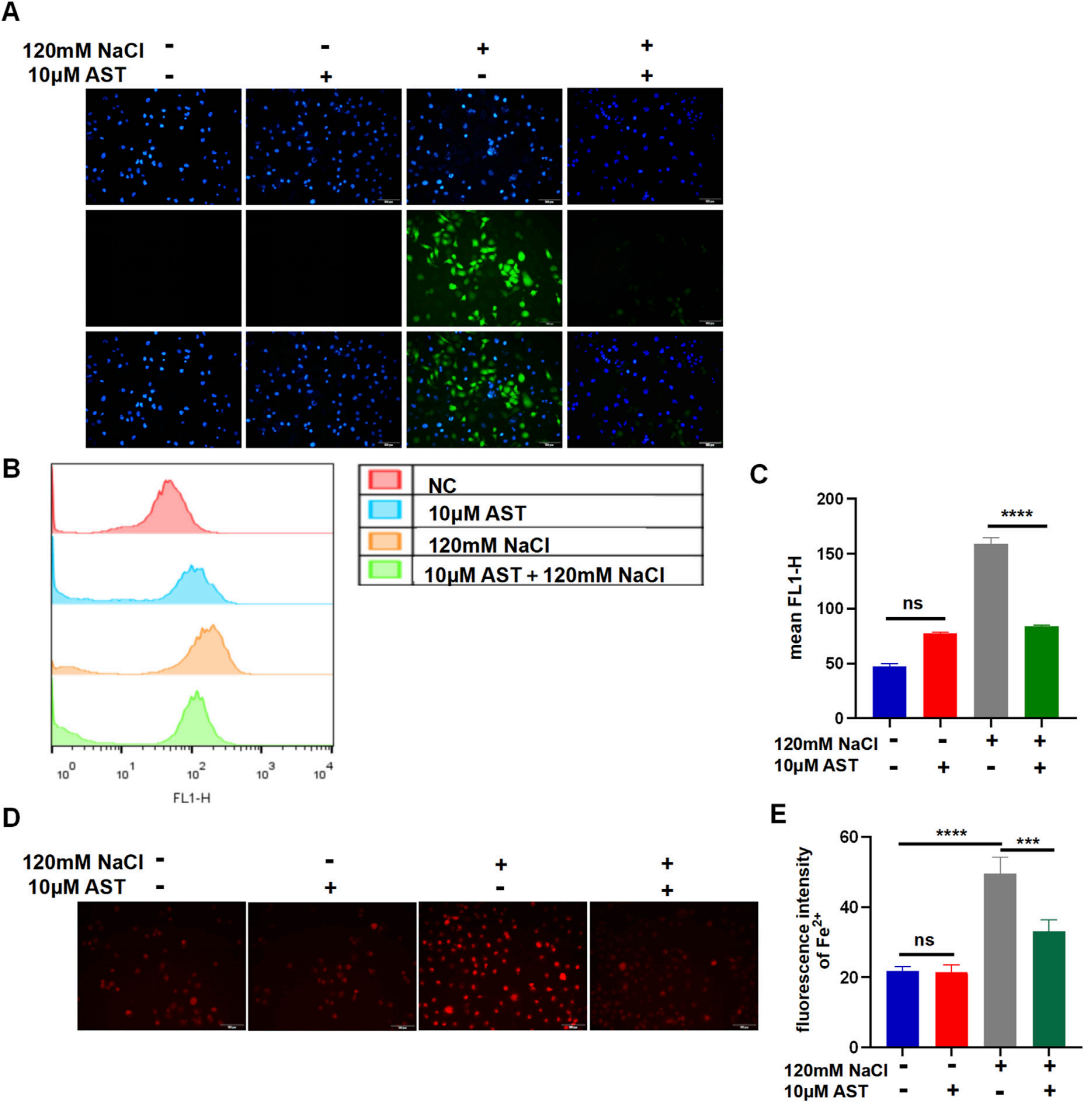
AST relieved hyperosmolarity-induced ferroptosis in HCECs. **(A)** The production of intracellular ROS was visualized using the fluorescent probe DCFH-DA and fluorescence microscopy. HCECs were pretreated with 10 μM AST for 6 h and then incubated with 120 mM NaCl for 24 h. Nuclei were stained blue with DAPI. Scale bar: 100 μM. **(B)** Quantification of fluorescence intensity of ROS acquired by flow cytometry. **(C)** Levels of ROS were stained by DCFH-DA with flow cytometry. **(D)** Intracellular Fe^2+^ was observed by fluorescence microscopy with 5 μM FeRhoNox-1. Scale bar: 100 μm. **(E)** Quantification of the fluorescence intensity of Fe^2+^ detected by FeRhoNox-1. ns, not significant; *p < 0.05, **p < 0.01, and ***p < 0.001.

The viability of HCECs with 120 mM NaCl for 24 h was improved from 48.93% to 87.24% by 10 μM AST and from 48.93% to 70.54% by 25 μM AST (Figure 5A). AST also remarkably increased the GSH level in hyperosmolarity-induced HCECs (Figure 5B). Western blot analysis also demonstrated that 10 μM AST increased protein levels of ferritin and GPX4 in HCECs and has no statistic influence on SLC7A11 (Figures 5C, D). The MDA levels in HCECs caused by treatment with 90 mM for 24 h were slightly but not significantly reduced by 10 μM AST (Figure 5E). However, 10 μM AST can remarkably relieve MDA caused by 120 mM NaCl (Figure 5E). Moreover, 10 μM AST significantly inhibited the production of ROS in HCECs after exposure to 120 mM NaCl (Figure 6A). Fluorescence quantization and flow cytometry results showed the same (Figures 6B, C). Confocal microscopy with FeRhoNox-1 staining showed that 10 μM AST significantly decreased the levels of Fe^2+^ induced by 120 mM NaCl in HCECs (Figures 6D, E). These results imply that AST may inhibit ferroptosis by upregulating GPX4 to protect hypertonic corneal epithelial cells.

### 3.6 Fer-1 and AST inhibited impairments of the mitochondrial ultrastructures

**FIGURE 7:**
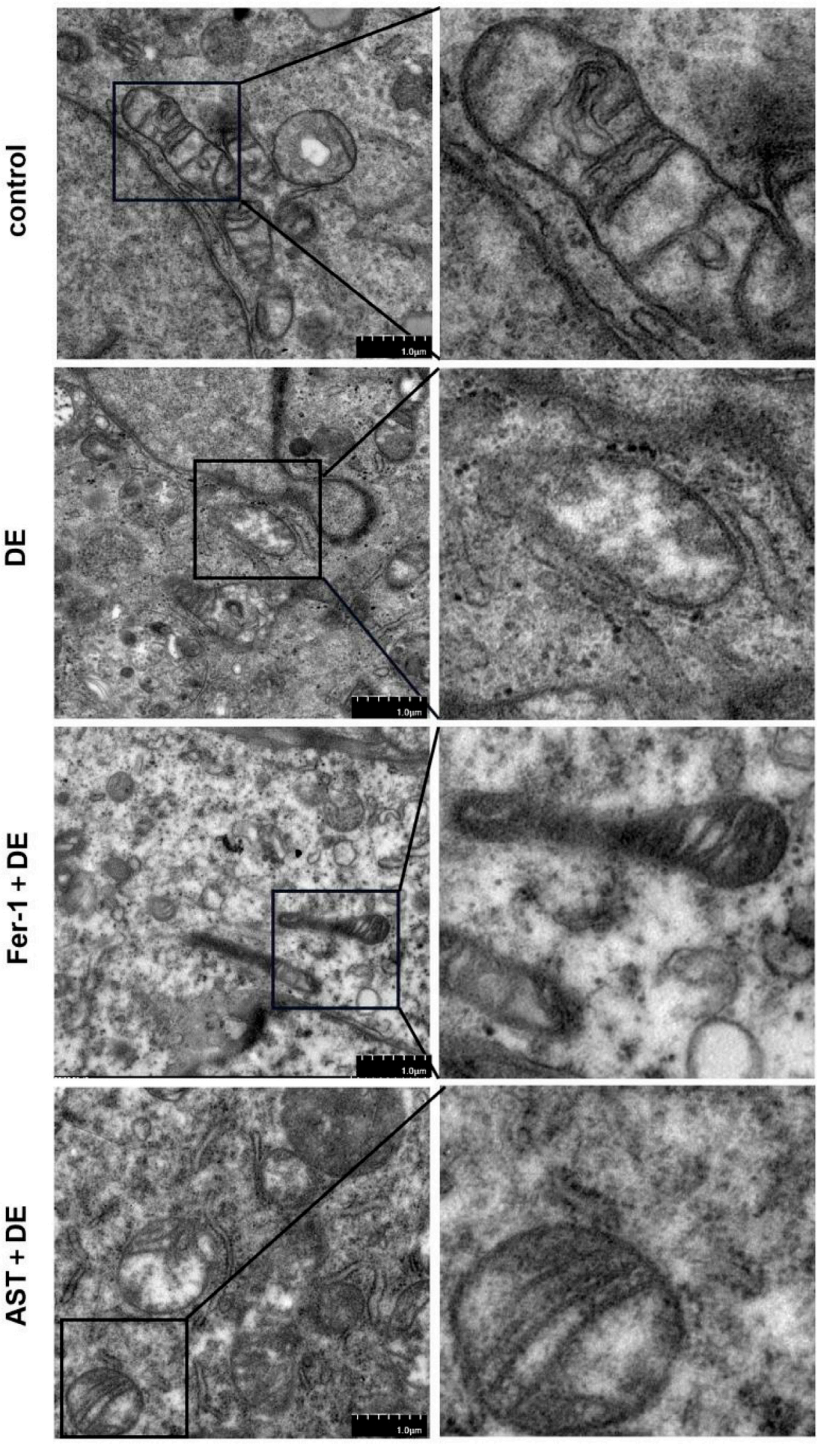
AST relieved the detrimental changes in mitochondrial ultra-structures. The ultra-structures of mitochondria were observed via transmission electron microscopy. The observation indicators included mitochondrial shrinkage, ruptured outer mitochondrial membrane, and reduced or disappeared mitochondrial cristae (DE indicates the dry eye group, treated with 120 mm NaCl).

We further investigated the alterations in mitochondria. Transmission electron microscopy demonstrated that mitochondria presented considerable shrinkage with a ruptured outer mitochondrial membrane and reduced or disappeared mitochondrial cristae, similar to morphological characteristics of ferroptosis, in hyperosmolarity-induced HCECs. However, Fer-1 and AST can restrain the detrimental damage to mitochondrial morphology (Figure 7).

### 3.7 Inhibition of ferroptosis can activate autophagy in hyperosmolarity-induced HCECs

**FIGURE 8:**
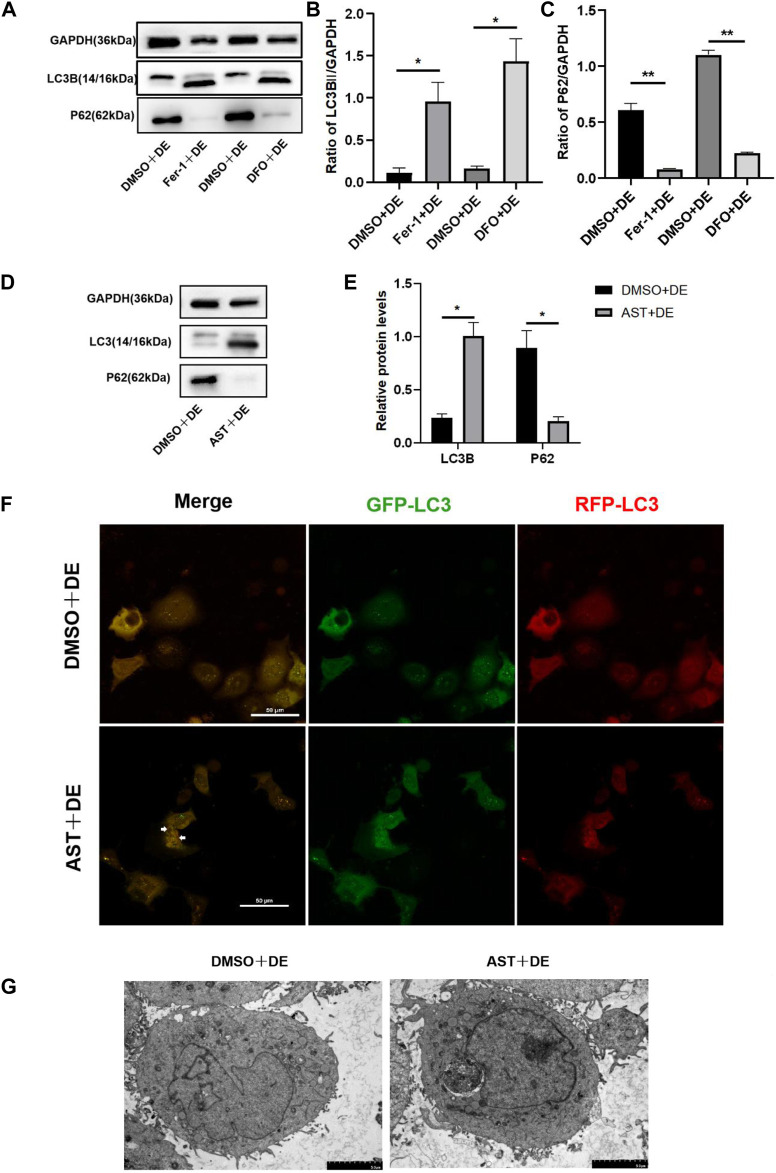
Inhibition of ferroptosis can activate autophagy in HCECs. **(A–E)** Western blot analysis of P62 and LC3B in HCECs after pretreating with DMSO, DFO, Fer-1, or AST for 6 h and then incubated for 24 h with 120 mM NaCl; protein levels were normalized to those of GAPDH. **(F)** The autophagic flow was detected by transfecting mRFP-GFP-LC3 under confocal microscopy. White triangles show red fluorescence. Scale bar: 50 μm **(G)**. Autophagosomes in HCECs were recorded by transmission electron microscopy. The scale bar was 5 μm; ns indicates not significant; *p < 0.05 and **p < 0.01 (DE indicates the dry eye group, treated with 120 mm NaCl).

Our previous literature reported that autophagy was not activated in HCECs treated with 120 mM NaCl (Pan et al., 2023). In the present study, Western blot results showed that the protein of LC3B increased and P62 reduced in hyperosmolarity-exposed HCECs pretreated with DFO, Fer-1, and AST compared with hyperosmolarity-exposed HCECs pretreated with DMSO (Figures 8A–E). The LC3 molecule is attached to autophagosomes, so its increase may be due to an increase in autophagosomes or a lack of degradation of autolysosomes. In order to know which one it is, HCECs were further infected with mRFP-GFP-LC3 to dynamically detect the state of autophagic flux. Under normal conditions, the autophagosome is labeled with yellow signals (mRFP and GFP), and after autophagosome–lysosome fusion, GFP is rapidly quenched by the low pH of lysosomes, and the autophagosome only appears as a red dot. In hyperosmolarity-induced HCECs pretreated with DMSO, we observed significant yellow fluorescence and no red dot, indicating that autophagic flux was inhibited. However, AST-protected cells showed yellow punctum reduction and red fluorescence appearance in the cytoplasm, revealing that AST recovered autophagic flux (Figure 8F). Similarly, TEM showed that the number of autophagosomes increased in HCECs in the AST + DE group compared with the DMSO + DE group (Figure 8G). Collectively, these results suggested that the inhibition of ferroptosis promotes autophagy in hyperosmolarity-exposed HCECs.

### 3.8 AST alleviates dry eye *in vivo*

**FIGURE 9:**
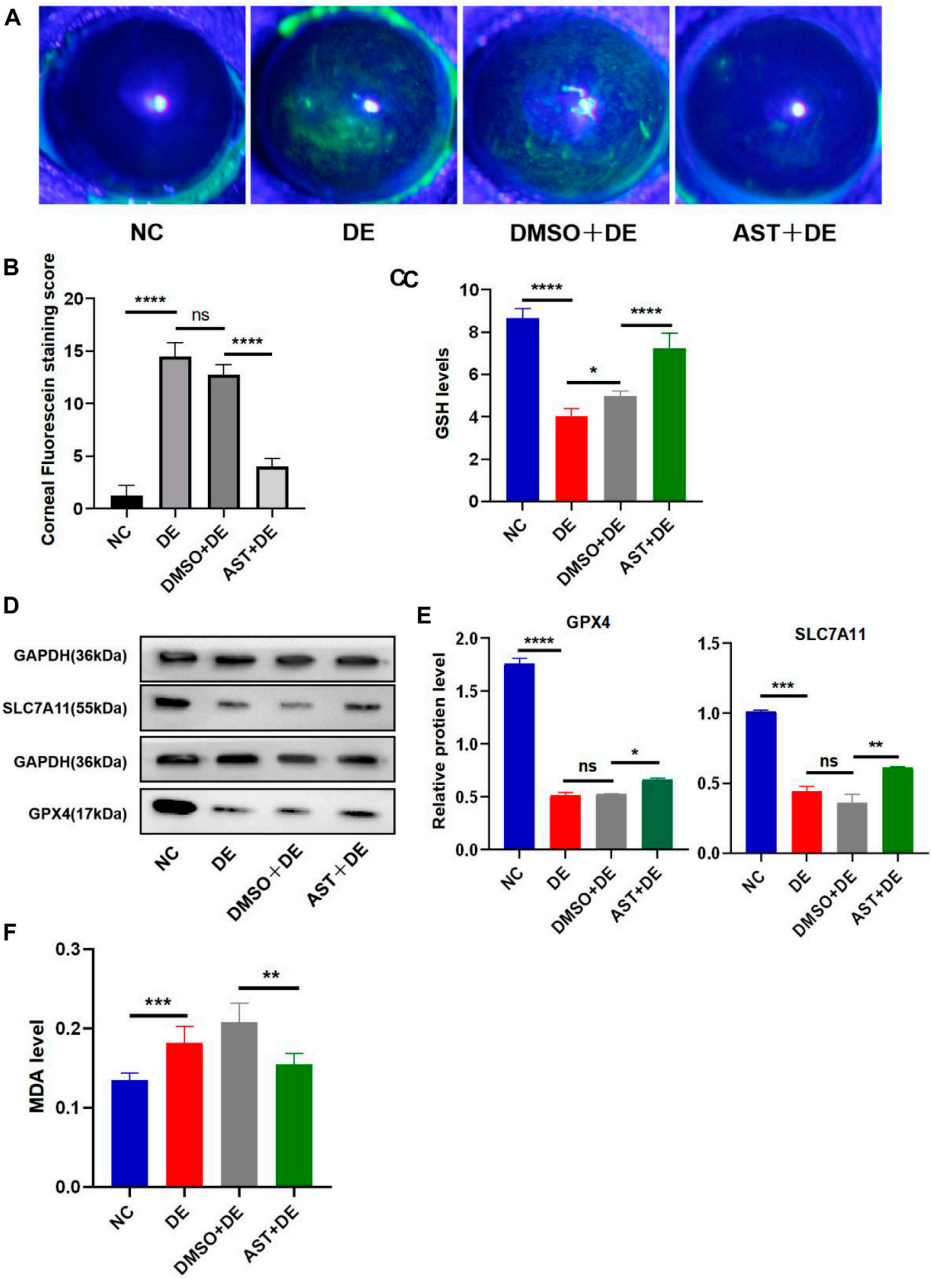
AST inhibits ferroptosis in the dry eye mouse model. **(A)** Photographs of corneal fluorescein staining. **(B)** Corneal fluorescein staining score. **(C)** GSH levels of the corneal tissue were examined using a GSH assay kit. **(D,E)** Western blot analysis of SLC7A11 and GPX4 in NC, DE, DMSO + DE, and AST + DE groups. GAPDH was used as the loading control. **(F)** GSH levels of corneal tissue were examined using a GSH assay kit. ns, not significant; *p < 0.05, **p < 0.01, ***p < 0.001, and ****p < 0.0001.

**FIGURE 10:**
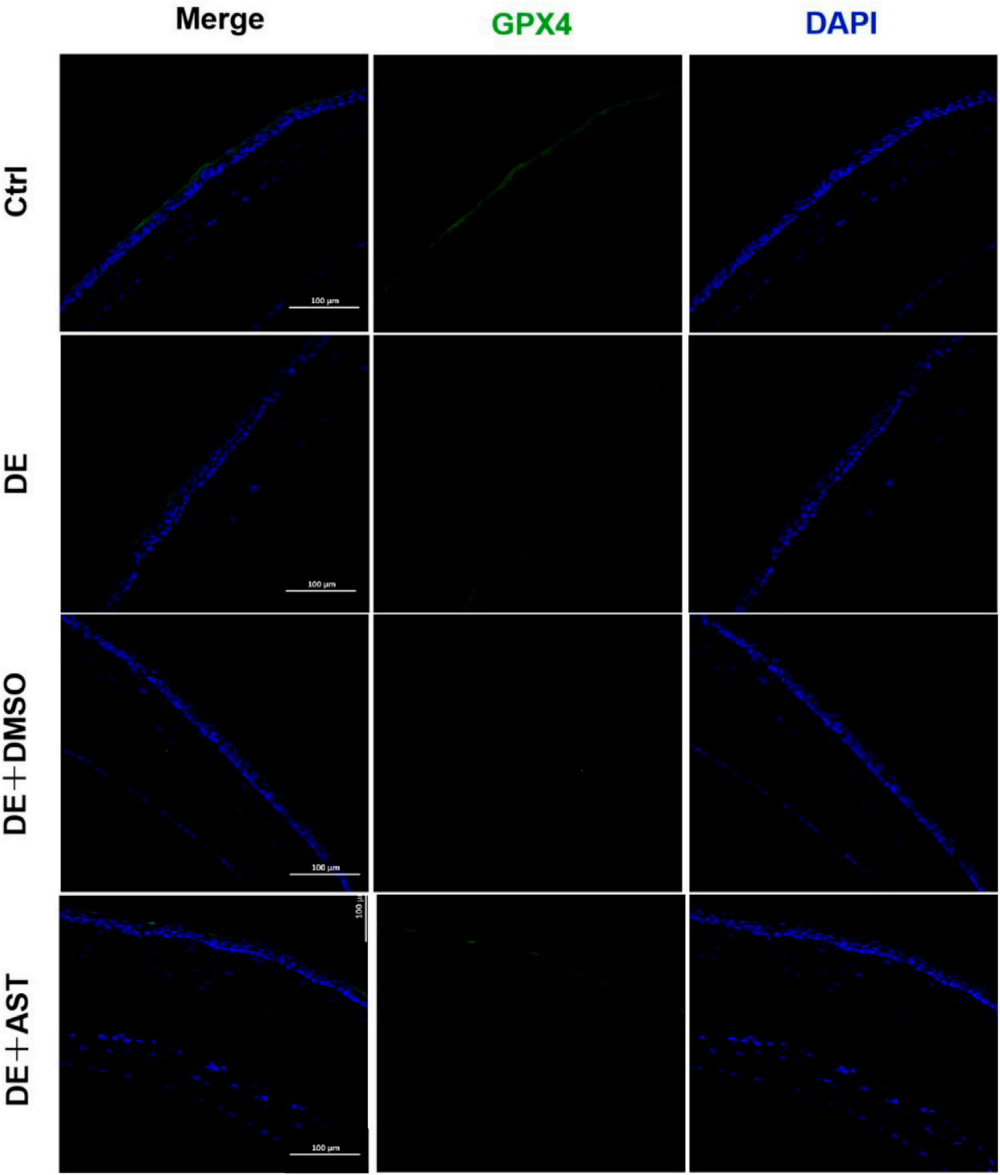
Representative fluorescence images showing the expression of GPX4 in the corneal epithelium of NC, DE, DMSO + DE, and AST + DE mice. Scale bar: 100 μm.

Previous studies have reported that ferroptosis occurred in the corneal epithelial cells in dry eye disease mice induced by scopolamine hydrobromide (Zuo et al., 2022). We established a dry eye disease mouse model to explore the effect of AST on ferroptosis *in vivo*. The corneal fluorescein staining score in the DE group was significantly higher than that in the control group, and AST rescued ocular surface defects (Figures 9A, B). The GSH expression of corneal tissue in the DE group was significantly lower than that in the control group. As expected, GSH levels in the AST-treated group improved (Figure 9C). Furthermore, it is found that the protein expression of SLC7A11 and GPX4 was downregulated in the DE and DMSO pretreated mice, and AST effectively increased both molecules, as demonstrated by Western blot and immunofluorescence staining (Figures 9D, E; Figure 10). MDA, considered a byproduct of lipid peroxidation (Yan et al., 2021), was used to indicate the content of lipid peroxidation *in vivo*. The MDA level was remarkably higher in the DE mice than that in the control mice, and AST decreased the MDA expression significantly compared with the DMSO + DE group (Figure 9F).

## 4 Discussion

**FIGURE 11:**
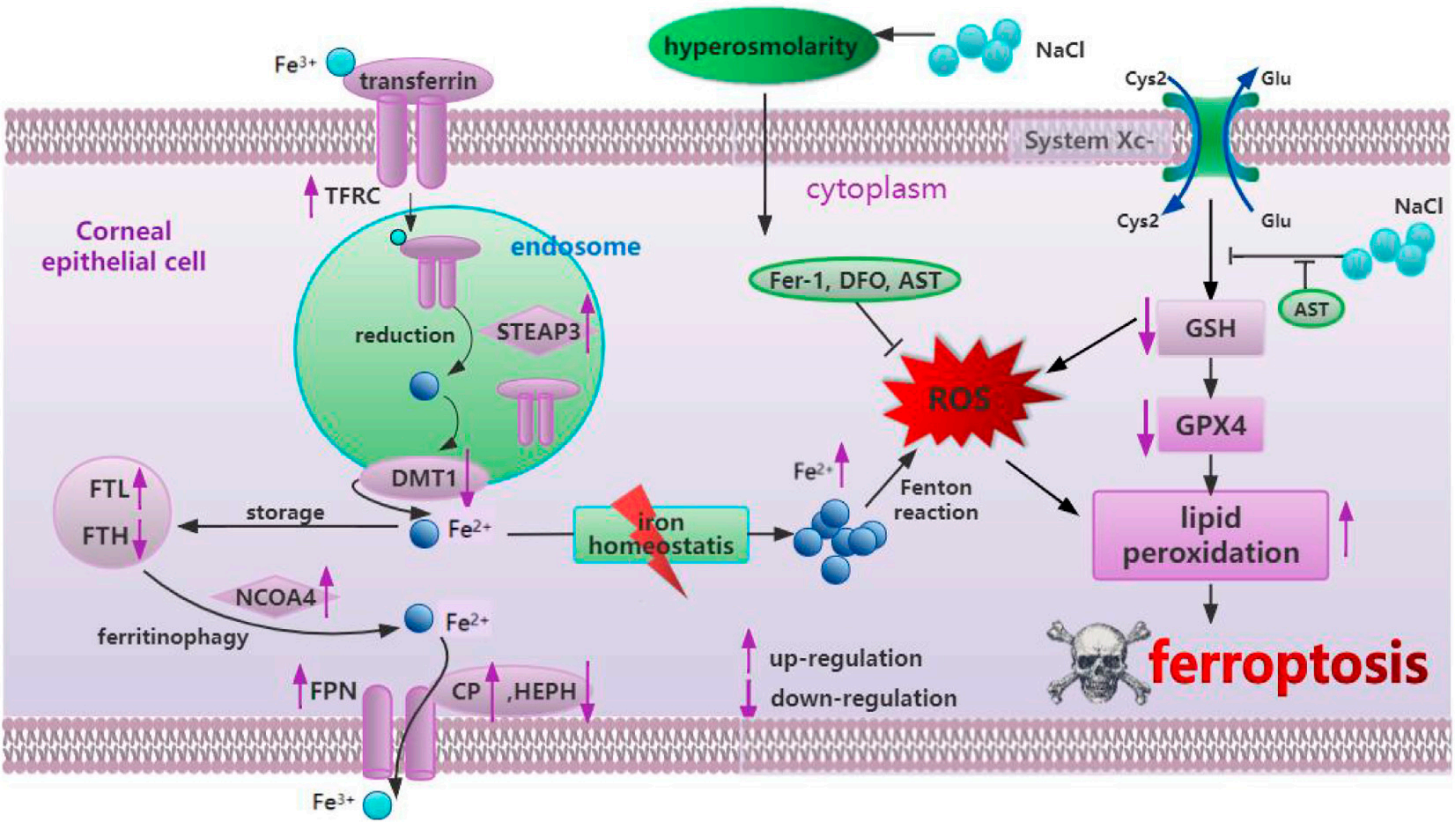
Summary of the mechanism of hyperosmolarity-induced ferroptosis in corneal epithelial cells and the protective effect of astaxanthin.

DED primarily originates due to hyperosmolarity in the tear film, resulting in ocular discomfort and even visual impairment, seriously affecting patient life quality (Baudouin et al., 2013). Previous studies have suggested that inflammation, cell necrosis, apoptosis, and autophagy play an important role in the pathogenesis of dry eye disease (Baudouin et al., 2013). Only one latest study found that ferroptosis occurred in dry eye by treating corneal epithelial cells with 94 mM NaCl *in vitro* and establishing a mouse model *in vivo* (Zuo et al., 2022), while our study provided additional evidence for ferroptosis by exploring iron metabolism-related and oxidative stress-related changes in corneal epithelial cells at different concentrations of NaCl (70, 90, and 120 mM). More importantly, in this study, it is strongly confirmed, for the first time, that AST could protect the mitochondrial structure, inhibit ferroptosis, and activate autophagy through the SLC7A11/GPX4 signaling pathway against DED *in vivo* and *in vitro* The effect of astaxanthin in hyperosmolarity-induced corneal epithelium cells was summarized in Figure 11.

Ferroptosis is characterized by the iron-dependent accumulation of lipid ROS and is morphologically and mechanistically distinct from other programmed cell death pathways (Dixon and Stockwell, 2014; Yang and Stockwell, 2016). In terms of morphology, ferroptosis appears mainly in cells as reduced mitochondrial volume, increased bilayer membrane density, and reduction or disappearance of mitochondrial cristae (Yang and Stockwell, 2008; Dixon et al., 2012). Previous studies have revealed that several substances can induce ferroptosis by different mechanisms (Liang et al., 2019; Wu et al., 2019). Although the physiological function of ferroptosis remains poorly defined, it has been shown to be involved in various diseases (Yuan et al., 2011; Coursey et al., 2016; Chen et al., 2020; Zuo et al., 2022). High concentrations of NaCl were used as our irritants to induce ferroptosis in HCECs. It should be mentioned here that the concentration of NaCl utilized in this study is of physiological significance (Totsuka et al., 2019). It is shown that 90 mM NaCl promotes the expression of inflammatory factors TNF- α, IL-1 β, and HMGB1 in corneal epithelial cells (Li et al., 2020). The key molecule of pyroptosis, N-GSDMD, was increased, and caspase-1 was upregulated by high-concentration NaCl (Li et al., 2022). Our results showed that 120 mM NaCl reduced cell viability, caused GSH depletion (Figure 2B), and GPX4 downregulation (Figures 2D–E). The normal synthesis of GSH relied on the abundant cysteine taken in the cell by system Xc-, which is distributed in phospholipid bilayers, composed of SLC7A11 and SLC3A2. We also indicated that intracellular ROS production enhanced in HCECs treated with increasing NaCl concentration until 120 mM showed statistical significance (Figures 2F–H). It was shown that 90 mM and 120 mM NaCl can remarkably improve lipid peroxide MDA generation (Figure 2C). As stated above, hyperosmolarity is likely to inhibit GSH synthesis instead of system Xc-, thereby decreasing the activity of GPX4, leading to a dysfunction in cell antioxidant capacity, accumulation of lipid ROS and peroxidation, and ferroptosis.

Previous studies demonstrated that iron overload gives rise to ROS by the Fenton reaction and elicits ferroptosis through ROS-mediated lipid peroxidation (Dixon et al., 2012). Intracellular Fe^2+^ levels have been shown to increase during ferroptosis in other cell types (Aron et al., 2016), which is consistent with our results, where intracellular Fe^2+^ levels increase in HCECs under hyperosmolarity-induced oxidative stress. Furthermore, we assessed changes in mRNA expression to clarify how several concentrations of NaCl affect iron homeostasis-regulating genes and revealed downregulation of HEPH and DMT1 and upregulation of FTL, CP, and FPN mRNA levels after NaCl exposure. These changes are considered to suppress intracellular Fe^2+^ levels, suggesting a compensatory response to increased Fe^2+^. In contrast, the upregulation of the mRNA level in NCOA4, TFRC, and STEAP3 and downregulation of FTH are regarded to increase cytosolic Fe^2+^ levels. The results confirmed that 120 mM NaCl interrupted iron homeostasis to increase Fe^2+^ levels (Figure 1) and induced lipid peroxidation, thus corroborating that hyperosmolarity activated corneal epithelial ferroptosis *in vitro* in an iron-dependent manner.

Fer-1 is a potent ferroptosis inhibitor that prevents the accumulation of lipid ROS. It has been shown that Fer-1 protects photoreceptor cells (Pan et al., 2023) and cancer cells from ferroptosis (Dixon et al., 2012; Yang and Stockwell, 2016; Chen C. et al., 2021). Here, the treatment of hyperosmolarity-loaded corneal epithelial cells with 10 μM Fer-1 remarkably increased cell viability via blocking ROS production, lipid peroxidation, and ameliorating intracellular Fe^2+^ overload (Figure 3). In addition, our results showed that iron chelator DFO also rescued cells from hyperosmolarity-induced death. Although most intracellular iron is tightly bound to proteins or incorporated into proteins as a cofactor or for storage, some free iron resides in the cytoplasm and intracellular organelles (such as lysosomes), constituting redox-active-dependent iron pools that regulate programmed cell death, including ferroptosis (Dixon and Stockwell, 2014). Since DFO is membrane-impermeable and accumulates in lysosomes, we propose that it protects the cells from ferroptosis by chelating lysosomal iron (Persson et al., 2003).

As the most powerful antioxidant, it is worth noting that the anti-ferroptosis mechanism of AST protecting DED has not been investigated *in vitro* and *in vivo*. The previous literature indicated that AST can ameliorate ferroptosis in acute lung injury and osteoarthritis patients (Luo et al., 2022; Wang et al., 2022). We found that AST can significantly improve the protein of GPX4 to reduce the inhibition of antioxidant capacity by hyperosmolarity. It also increased the ferritin content to enhance intracellular iron storage and decrease the Fe^2+^ overload. Ultimately, the accumulation of ROS and MDA in HCECs was scavenged effectively. Simultaneously, *in vivo*, AST could lessen ferroptosis through boosting important ferroptosis-related markers GSH, SLC7A11, and GPX4 (Figures 9C–F; Figure 10). In addition, mitochondria may be important sub-cellular organelles targeted for ferroptosis. Evidence suggests that mitochondrial involvement in ferroptosis is closely related to the function of classical mitochondrial metabolic activities, possibly due to the mitochondrial tricarboxylic acid (TCA) cycle and mitochondrial electron transport chain (ETC), promoting intracellular ROS and lipid ROS production (Gao et al., 2019). SLC25A37 and SLC25A28 mediate intracellular iron transport to mitochondria. Both impaired intracellular iron and mitochondrial iron homeostasis are considered hallmarks of ferroptosis and promote the ferroptosis process (Huang et al., 2021). Fer-1 and AST remedied the harmful effects of mitochondria in hyperosmolarity-struck ferroptosis, including outer membrane rupture and vacuolation, reduced mitochondrial volume, and reduced or absent mitochondrial cristae (Figure 7).

Currently, autophagy is considered to be a cellular defense and stress regulation mechanism to maintain homeostasis under stress conditions. Additionally, there is a decrease in autophagy in some diseases, such as cancer and neurodegeneration (Filomeni et al., 2010; Dikic and Elazar, 2018). AST has been found to increase autophagy flux and inhibit ferroptosis to attenuate liver injury (Cai et al., 2022). As for DED, our previous research acknowledged that AST suppresses inflammation by downregulating the expression of HMGB1 and inflammation in DED models via the PI3K/Akt signaling pathway (Li et al., 2020). In the present study, the results provide sufficient evidence for elucidating the relationship between ferroptosis and autophagy in DED. Our experimental data originally suggested that AST could inhibit ferroptosis via the SLC7A11/GPX4 pathway and promote the generation of healthy autophagic flux to relieve DED. A previous study suggested that excessive oxidative stress-induced impaired autophagic flux is a key pathogenic event in DED (Wang et al., 2021). ROS are early inducers of autophagy. Fer-1, DFO, or AST can effectively scavenge ROS, thereby activating normal autophagy.

In summary, we provide complementary data on ferroptosis in dry eye disease. More importantly, we revealed, for the first time, that AST efficiently protected corneal epithelial cells against ferroptosis in DED both *in vitro* and *in vivo*. AST extensively reduced excessive ROS production, rescued the mitochondrial structure via the SLC7A11/GPX4 pathway, and restored the defected autophagic flux in HCECs under hyperosmolarity. Lastly, our study suggested that the cytoprotective function of AST relied on redox regulation. Thus, these data, for the first time, provide a novel therapy for DED treatment and elaborate a better understanding of its pathogenesis.

## Data Availability

The original contributions presented in the study are publicly available. The immunofluorescence data can be found at: https://figshare.com/articles/figure/figures/26763082. Further enquiries can be directed to the corresponding author.
