# Supplementary figures and images for: Astaxanthin activated the SLC7A11/GPX4 pathway to inhibit ferroptosis and enhance autophagy, ameliorating dry eye disease
Source: Front Pharmacol. 2024 Aug 19;15:1407659. doi: 10.3389/fphar.2024.1407659 (PMC11366873; doi:10.3389/fphar.2024.1407659)

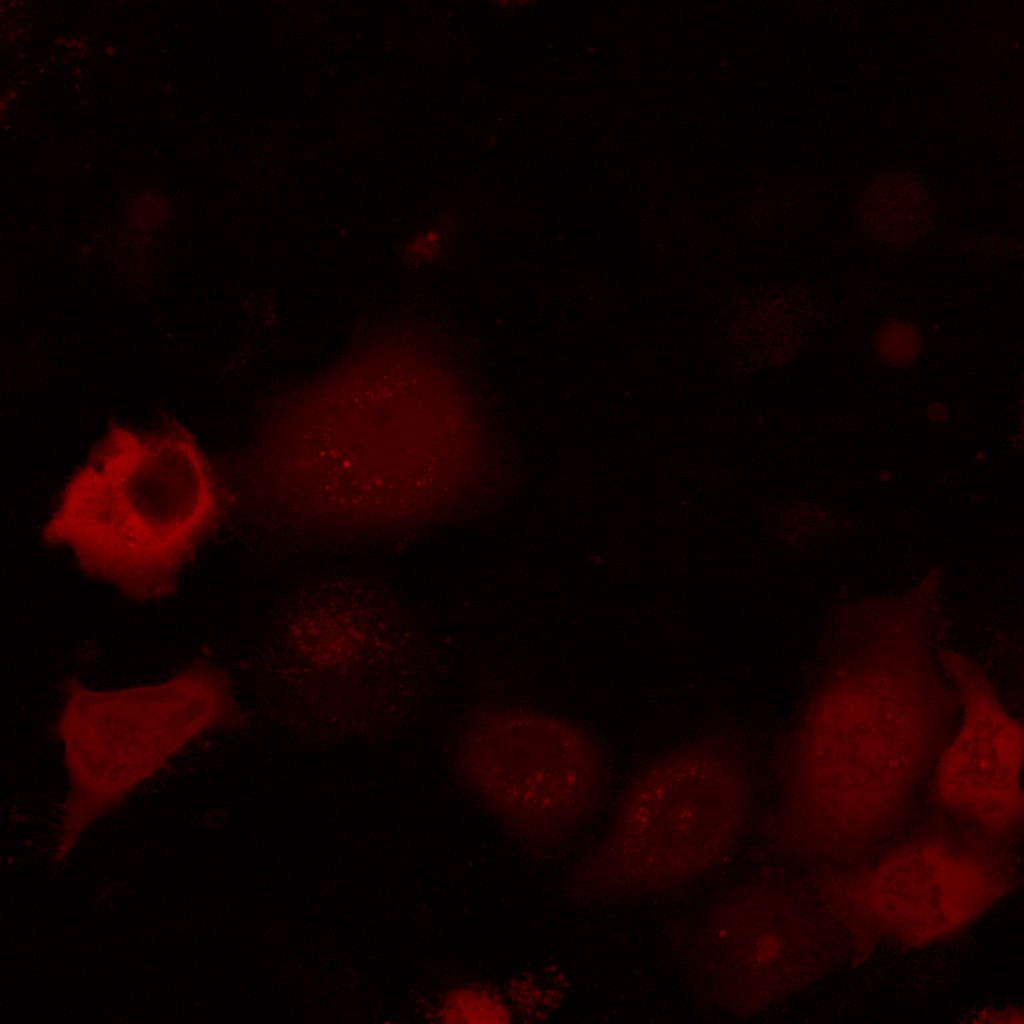

Supplement: Supplementary file 1 [file Image6.TIF]

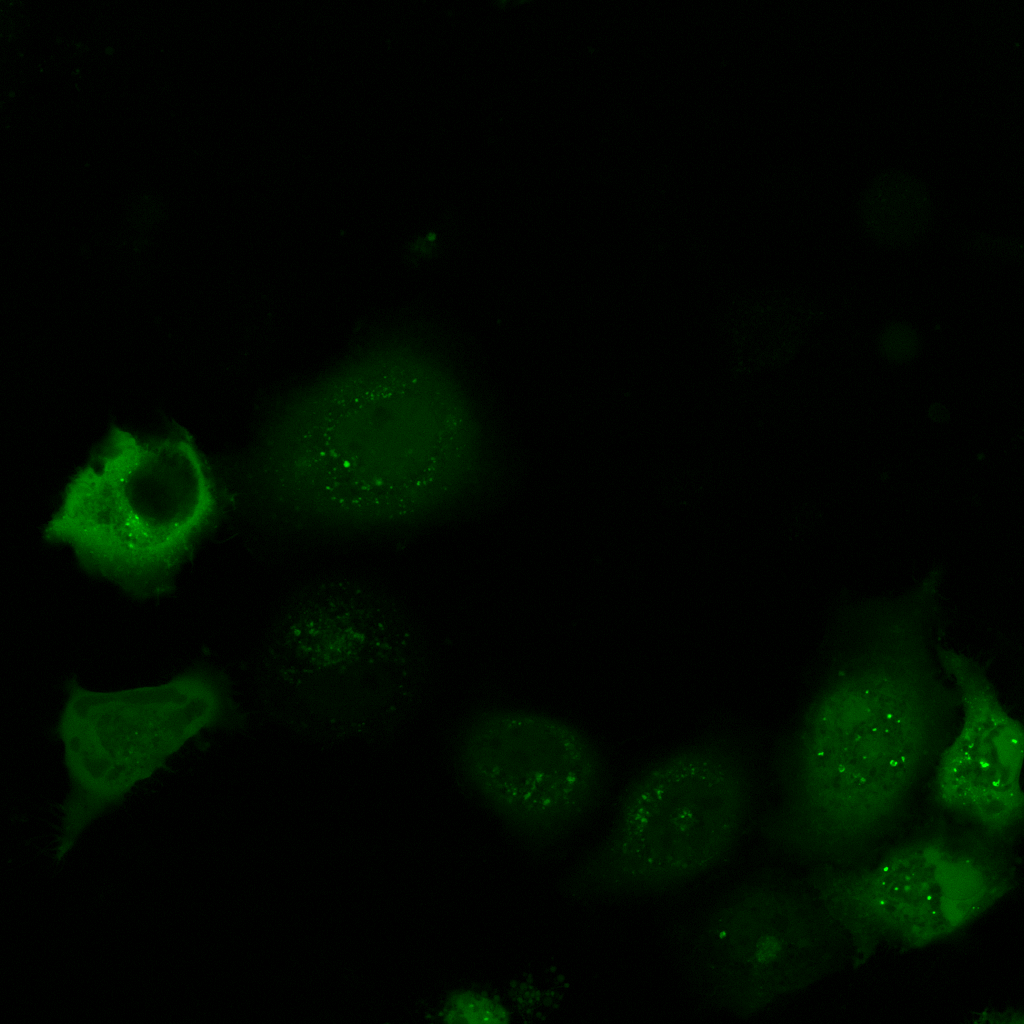

Supplement: Supplementary file 2 [file Image3.TIF]

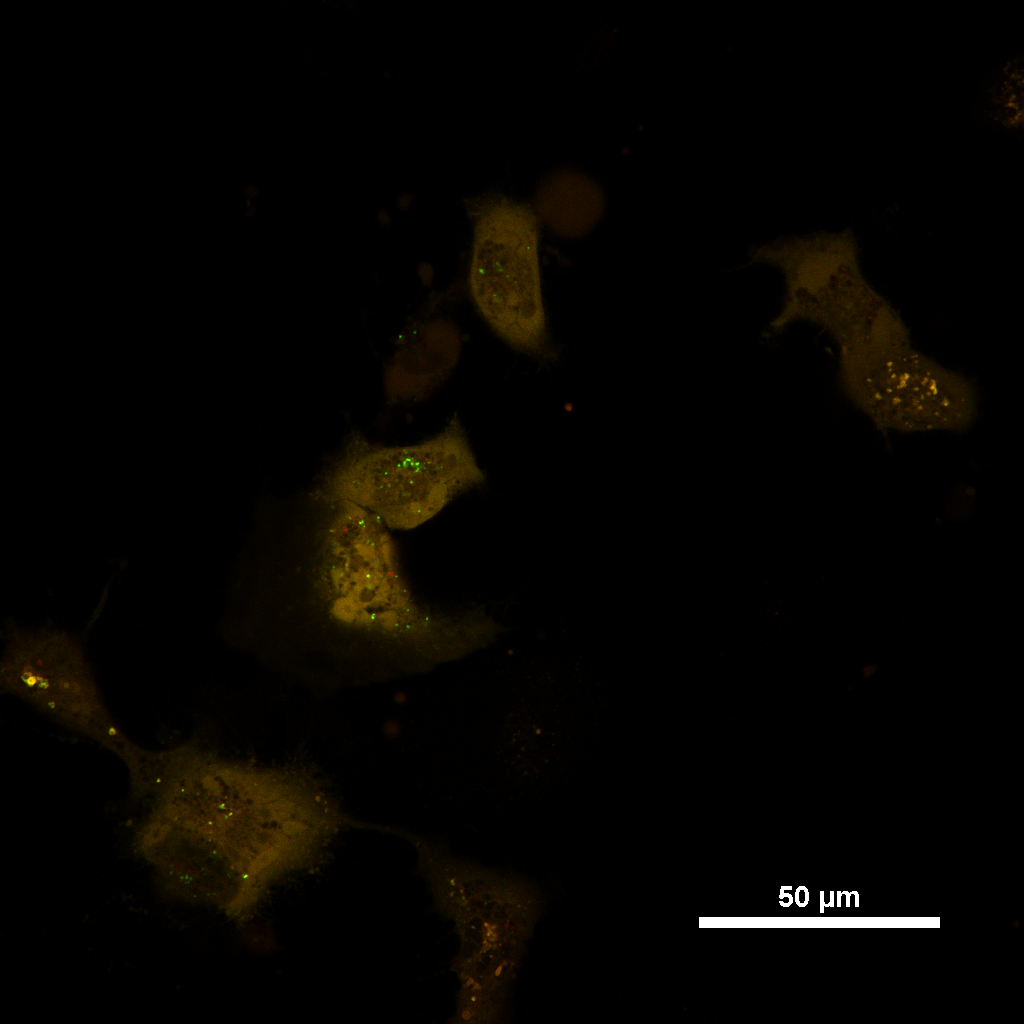

Supplement: Supplementary file 3 [file Image4.TIF]

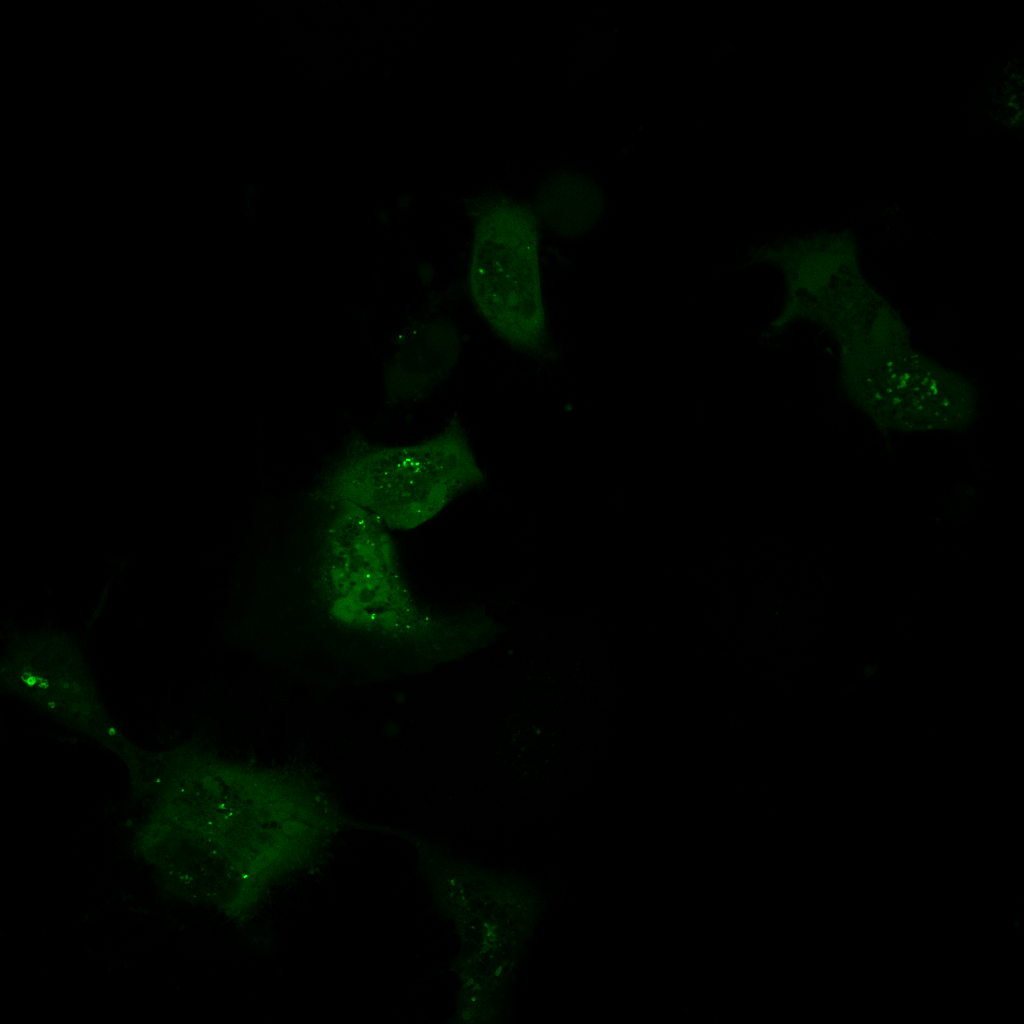

Supplement: Supplementary file 4 [file Image2.TIF]

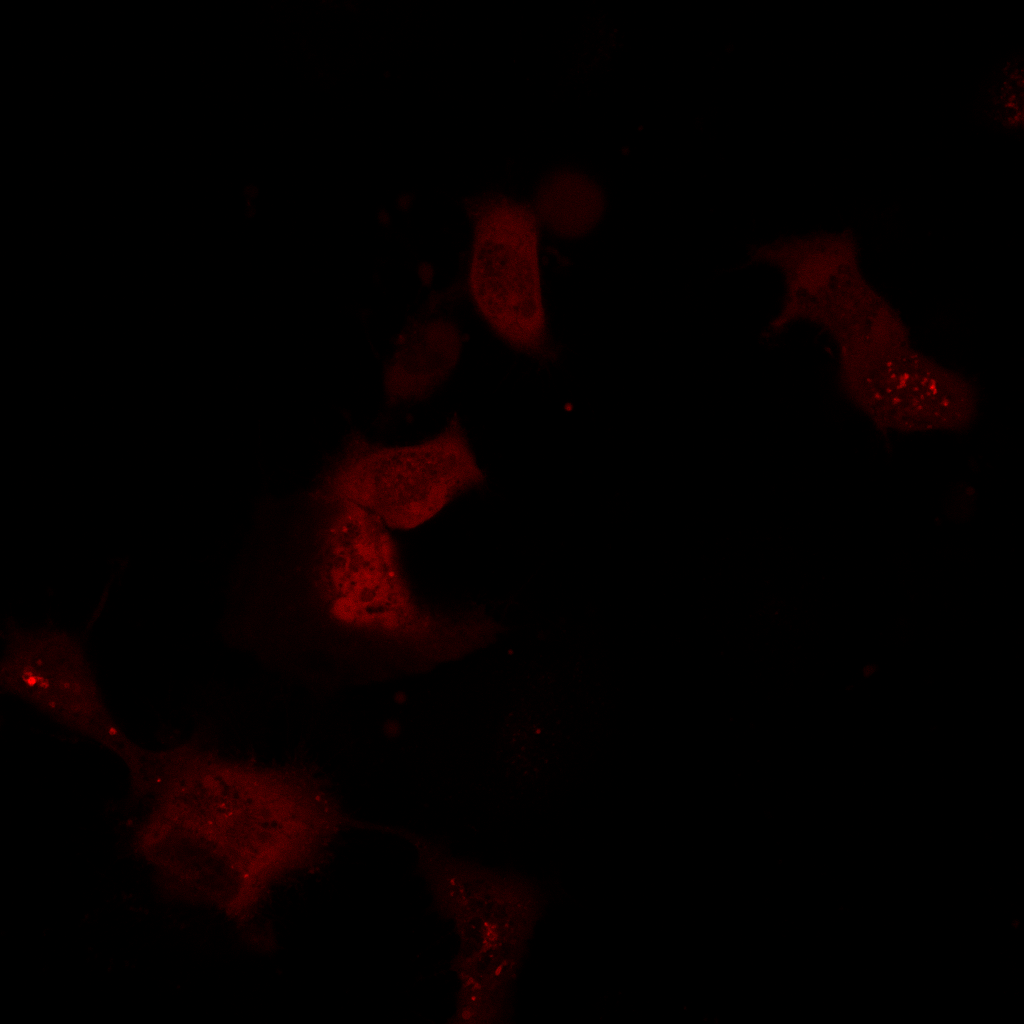

Supplement: Supplementary file 5 [file Image1.TIF]

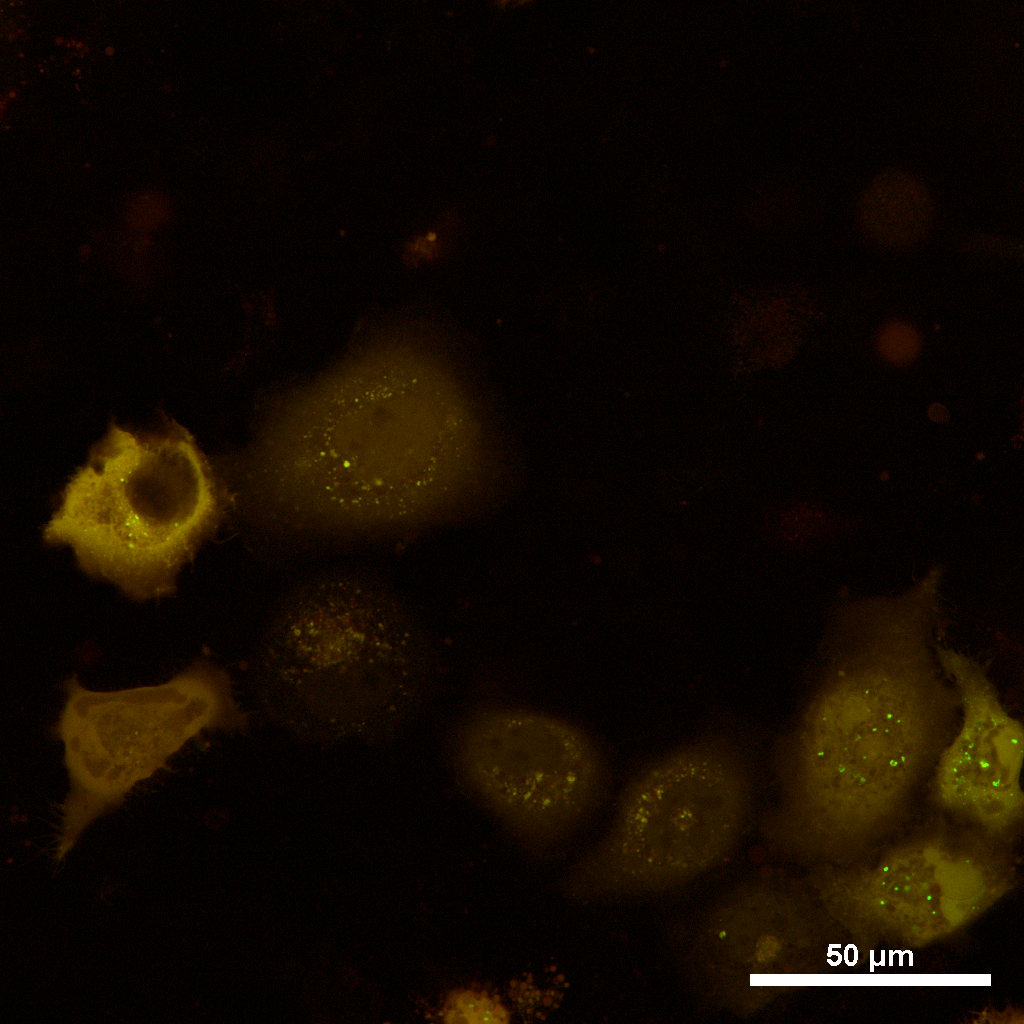

Supplement: Supplementary file 7 [file Image5.TIF]
